# Single‐Atom Intercalation‐Driven Topological Ferroelectric Metal for High‐Performance Hydrogen Evolution Reaction

**DOI:** 10.1002/advs.75741

**Published:** 2026-05-19

**Authors:** Rongxuan Lu, Jian Zhang, Jialin Gong, Wenxian Li, Gang Zhang

**Affiliations:** ^1^ School of Chemical Engineering University of New South Wales Sydney New South Wales Australia; ^2^ Yangtze Delta Region Academy in Jiaxing Beijing Institute of Technology Jiaxing China; ^3^ School of Materials Science and Engineering Beijing Institute of Technology Beijing China; ^4^ Institute for Superconducting and Electronic Materials Faculty of Engineering and Information Sciences University of Wollongong Wollongong New South Wales Australia

**Keywords:** ferroelectric Weyl metal, HER, single‐atom intercalation

## Abstract

Intercalation engineering has become an effective strategy for tailoring the electronic and structural properties of layered materials, enabling functionalities that are absent in their pristine counterparts. In this work, we propose that intercalation engineering can be used to realize multifunctional catalysts that combine metallic and topological electronic structures with switchable polarization and catalytic activity—an outstanding challenge in materials science. Specifically, inserting an isolated Cu atom into monolayer AB_3_ (A = Bi, Sb, As; B = Cl, Br, I) converts the parent nonpolar semiconductors into ferroelectric (FE) metallic systems with switchable FE polarization. This intercalation simultaneously reconstructs the electronic band structures and induces Weyl points near the Fermi level. Remarkably, the presence of an intercalated Cu atom substantially enhances the intrinsic hydrogen evolution reaction (HER) activity of monolayer AB_3_, leading to a significantly reduced hydrogen adsorption free energy. Furthermore, the switchable FE polarization enables effective modulation of the catalytic activity, allowing the HER performance to be tuned by FE polarization reversal. Our results establish single‐atom intercalation as a powerful route to couple ferroelectricity, topological metals, and electrocatalysis, opening a pathway toward electrically controllable topological FE catalysts for sustainable hydrogen production.

## Graphical abstract

Single‐atom Cu intercalation enables porous AB_3_ monolayers to integrate ferroelectricity, metallicity, topology, and hydrogen evolution catalysis in one platform. Switchable FE polarization modulates work function and hydrogen adsorption thermodynamics, providing an electrically controllable route to activate basal‐plane HER activity in topological ferroelectric metals.

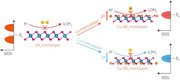

## 1 Introduction

2D materials have emerged as attractive candidates for electrocatalysis owing to their atomic thickness, large specific surface area, and readily engineerable electronic structures [1, 2, 3]. These features offer opportunities to modulate adsorption energetics and charge transfer processes that govern hydrogen evolution reaction (HER) kinetics. Nevertheless, the catalytic potential of most pristine 2D materials is not realized on the basal plane, where saturated coordination and semiconducting character suppress charge redistribution and weaken the binding of reaction intermediates. For example, monolayer MoS_2_ has long served as a prototypical HER electrocatalyst, owing to the high activity of its metallic edge sites, which exhibit a nearly thermoneutral Gibbs free energy of hydrogen adsorption (Δ*G*_H*_ ≈ 0.06 eV), whereas hydrogen adsorption on the basal plane is thermodynamically unfavorable, with Δ*G*_H*_ as high as ≈1.92 eV [4, 5, 6]. Consequently, HER activity in many 2D materials is often confined to edges, vacancies, or other defects [7, 8, 9, 10, 11]. While defect/edge engineering can enhance activity, it typically provides a limited density of active sites and compromises structural uniformity, making deterministic and reversible regulation challenging. Therefore, a key goal is to activate the basal plane in a controllable manner—ideally by simultaneously reshaping lattice symmetry and electronic states to enable efficient charge transfer and optimal H* adsorption.

In this context, intercalation engineering has emerged as a powerful and versatile strategy for activating the basal plane of 2D materials, as it enables the concurrent modulation of lattice symmetry and electronic structure at the atomic scale [12, 13, 14, 15, 16, 17, 18, 19, 20, 21]. By inserting a single atom into layered or porous lattices, intercalation induces pronounced charge redistribution and local structural asymmetry while preserving the overall crystallographic framework. From an experimental perspective, single‐atom intercalation, particularly Cu intercalation, may be achievable through several routes that have been widely explored in layered and porous materials, including electrochemical intercalation [22], solution‐assisted ion insertion [23], and self‐intercalation during growth [24]. From a structural perspective, the inserted atoms locally reconstruct the coordination environment of surrounding atoms, giving rise to asymmetric lattice relaxation manifested as modified bond lengths, bond angles, interlayer spacing, and site symmetry [25, 26]. These local distortions break the equivalence of crystallographic sites and effectively lower the symmetry of the pristine lattice, while maintaining overall structural coherence and uniformity across the basal plane. Such symmetry reduction provides an essential structural basis for accessing lattice configurations and interfacial environments that are inaccessible in the pristine host. From an electronic perspective, strong hybridization between the intercalant and host states can substantially reconstruct the electronic structure, particularly near the Fermi level. In many cases, this hybridization leads to the emergence of new electronic states or even drives a semiconductor–metal transition, accompanied by enhanced carrier density and improved charge‐transport capability. These electronic changes directly address one of the key limitations of pristine 2D basal planes, namely their insufficient charge supply and poor electronic conductivity. By simultaneously reshaping the local bonding environment and the electronic landscape of the basal plane, intercalation engineering establishes a coherent framework for tuning surface reactivity in a controllable and spatially uniform manner. Meanwhile, an increasing number of studies have indicated that single‐atom adsorption or intercalation can also be exploited to induce and tune ferroic responses in 2D systems. Depending on the host material and incorporation mode, such single‐atom engineering has been reported to generate out‐of‐plane polarization, multiple ferroelectric (FE) states, and even coupled FE–magnetic behavior [27, 28]. Despite these advances, the simultaneous realization of intercalation‐induced metallic ferroelectricity and polarization‐controlled basal‐plane HER in a single 2D system remains largely unexplored.

In this work, we demonstrate that single‐atom intercalation provides a unified materials‐design strategy for simultaneously activating ferroelectricity, topological metallic states, and high HER activity in 2D systems. Specifically, intercalation of an isolated Cu atom into monolayer AB_3_ (A = Bi, Sb, As; B = Cl, Br, I) transforms the parent centrosymmetric nonpolar semiconductors into metallic ferroelectrics with reversible out‐of‐plane polarization. Cu intercalation not only breaks inversion symmetry and stabilizes FE order, but also reconstructs the electronic band structures, giving rise to Weyl points in the vicinity of the Fermi level. Remarkably, compared with pristine AB_3_ monolayers, the Cu‐intercalated systems exhibit substantially optimized hydrogen adsorption thermodynamics, indicating a pronounced activation of basal‐plane HER activity. Moreover, the switchable FE polarization enables modulation of H* binding strength, thereby allowing HER performance to be reversibly tuned. These findings establish single‐atom intercalation as a powerful route to couple ferroic order, topological metallic states, and electrocatalysis, and open a pathway toward electrically controllable topological catalysts for sustainable hydrogen production.

## 2 Results and Discussion

### 2.1 Structural Characteristics and Poor HER Activity of Monolayer AB_3_

To identify target materials with optimal properties, we conducted a systematic high‐throughput screening of the Computational 2D Materials Database (C2DB). In this screening, we focused on monolayers that are structurally stable, exhibit semiconducting behavior, and possess periodic pores within the lattice to serve as favorable hosting sites for atomic intercalation. This screening process ultimately identified AB_3_ (i.e., AsI_3_, SbI_3_, BiI_3_, BiCl_3_, and BiBr_3_) monolayers as promising candidates. Structurally, all the monolayers crystallize in the centrosymmetric layer group (LG) 71, where each A atom is coordinated by six slightly distorted B atoms. Owing to their analogous electronic configurations, all AB_3_ materials exhibit essentially identical electronic characteristics. Electronic band‐structure calculations confirm that all AB_3_ monolayers are semiconductors, as shown in Figure S1.

We further evaluated the HER catalytic performance of pristine AB_3_ monolayers by examining four representative adsorption sites, including one hollow site (S1) and three atop sites (S2–S4), as illustrated in Figure S2. The calculated Gibbs free energies for hydrogen adsorption (Δ*G*_H*_) at these sites for all AB_3_ monolayers are summarized in Table S1. Notably, the calculated ΔG_H*_ values for all considered sites deviate significantly from zero, indicating that the pristine surfaces cannot effectively bind H* and are therefore intrinsically inactive for HER on the basal plane. This largely originates from the semiconducting and coordination‐saturated nature of the AB_3_ basal planes. Specifically, the low density of electronic states near the Fermi level, together with the absence of unsaturated surface bonds, suppresses charge transfer and weakens H* binding, leading to large positive Δ*G*_H*_ (1.673–2.861 eV).

### 2.2 Cu‐Intercalated AB_3_ Monolayers: Topological Ferroelectric Metals with Switchable HER Activity

A practical route to improve basal‐plane HER activity in AB_3_ monolayers is to engineer local electronic environments via single‐atom intercalation, which can optimize hydrogen binding and enhance the HER catalytic performance. In this regard, copper (Cu) is selected as a prototypical intercalant, as it is known to enhance carrier mobility [24] and induce FE polarization [27, 28, 29]. However, single‐atom intercalation in porous AB_3_ monolayers can in principle occur at multiple nonequivalent sites, leading to distinct local coordination environments and, consequently, different electronic states and physical properties. To make the search tractable and physically meaningful, three criteria were imposed to identify the most relevant intercalation sites. First, to ensure the stability of the active site, the Cu atom should be steadily adsorbed on the surfaces of AB_3_ monolayers. Second, the preferred adsorption site should remain essentially unchanged upon FE polarization switching; Third, after intercalation, the resulting Cu‐AB_3_ monolayers should be capable of activating hydrogen adsorption and maintaining ferroelectricity. After a comprehensive evaluation, the C hollow site was identified as the most promising adsorption site for Cu intercalation and was therefore selected for further investigation. The other three sites either fail to maintain a switchable FE modulation or cannot effectively activate H* (see Table S2 and Figure S5).

Building on this site selection, we next examine the electronic and structural characteristics of Cu intercalation at the C hollow site. First, Cu intercalation breaks the inversion symmetry of the AB_3_ monolayers, transforming the structure from the centrosymmetric nonpolar LG 71 to the polar LG 70 and thereby inducing a switchable out‐of‐plane FE polarization. Second, the Cu‐AB_3_ system undergoes a semiconductor‐to‐metal transition, accompanied by a pronounced enhancement of the density of states near the Fermi level E_f_. Importantly, within this reconstructed metallic band structure, Weyl points with Fermi velocity comparable to that of graphene are identified in the vicinity of E_f_, which are expected to exert a profound influence on the HER process.

**FIGURE 1:**
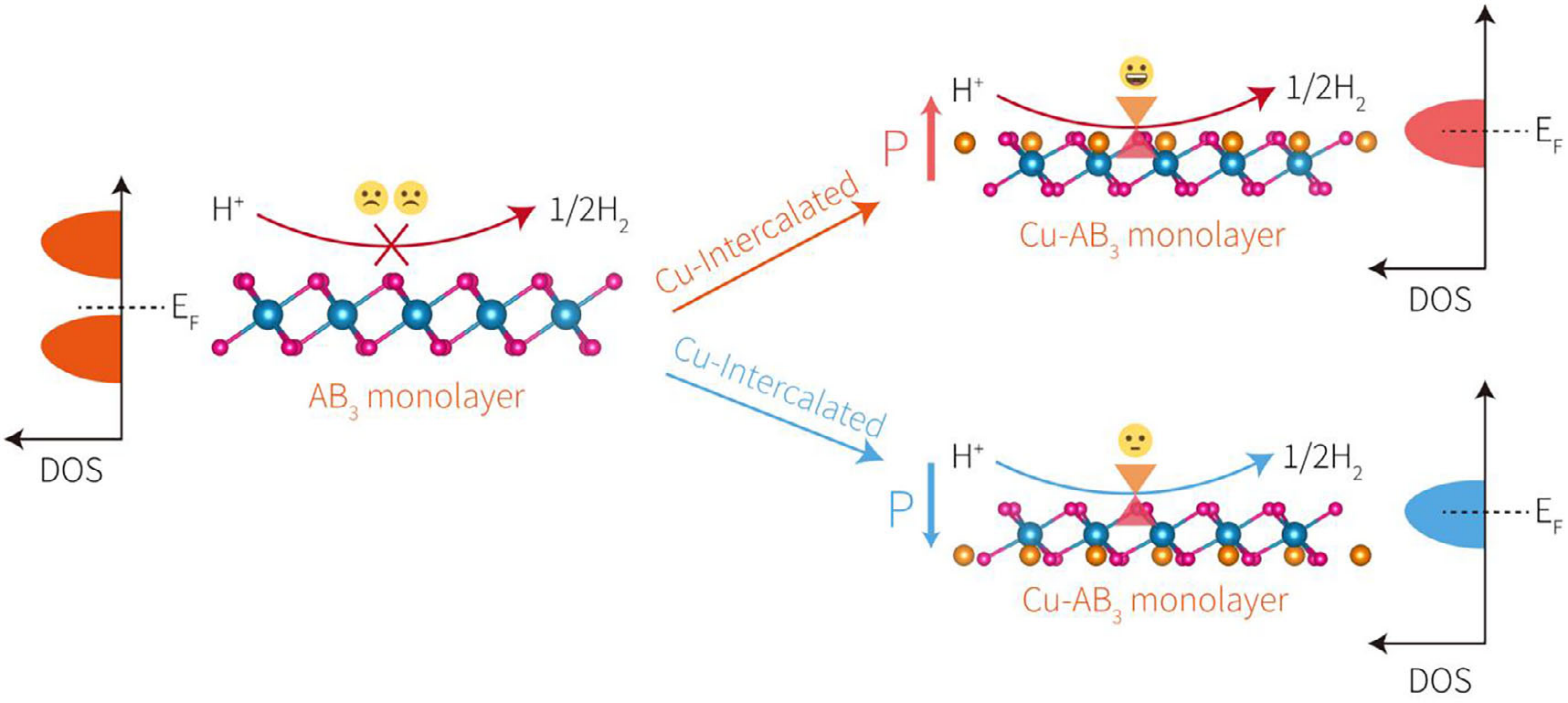
Schematic illustration of Cu‐intercalation‐induced ferroelectricity, metallicity, Weyl points, and switchable HER activity in AB_3_ monolayers.

Taken together, Cu intercalation at the C site simultaneously (i) induces ferroelectricity, (ii) drives metallization, (iii) generates band crossing features near E_f_, and (iv) enables FE modulation of catalytic activity, as schematically illustrated in Figure 1. The stability of the Cu‐intercalated structures was further confirmed by ab initio molecular dynamics (AIMD) simulations at finite temperature and phonon dispersions (see Figures S3 and S4 for details).

#### 2.2.1 Ferroelectricity of Monolayer Cu‐BiCl_3_

**FIGURE 2:**
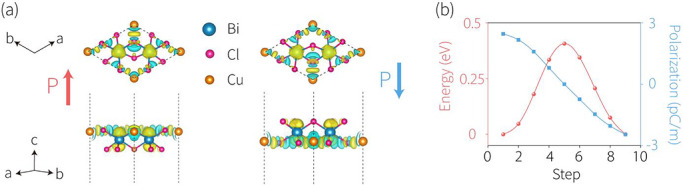
(a) Charge‐density difference of Cu–BiCl_3_ in the P↑ and P↓ FE states. (b) Energy barrier (red) and polarization (blue) evolution along the FE switching pathway of Cu‐BiCl_3_.

To gain deeper insight into the microscopic mechanism induced by single‐atom intercalation, Cu‐BiCl_3_ is taken as a representative example. As shown in Figure 2a, the charge‐density difference clearly reveals pronounced charge redistribution around the intercalated Cu atom and its neighboring Bi and Cl atoms in both P↑ and P↓ states. Reversal of the FE polarization leads to an asymmetric redistribution of the charge‐density along the out‐of‐plane direction, indicating a strong coupling between Cu atoms and the local bonding environments. Correspondingly, the FE switching pathway, displayed in Figure 2b, exhibits a moderate energy barrier, accompanied by a continuous evolution of polarization, confirming the robust switchability of the FE state induced by Cu intercalation. These results demonstrate that Cu insertion not only induces ferroelectricity, but also enables reversible polarization switching without structural instability.

Specifically, the energy barrier along the FE transition path is calculated to be 0.407 eV per unit cell, which is lower than those reported for monolayer SiN (0.70 eV per unit cell) [30] and single‐layer FeO_2_H (0.457 eV per unit cell) [31], suggesting that Cu‐intercalated BiCl_3_ can undergo electric‐field‐induced switching under practical conditions. The out‐of‐plane electric polarization is defined as

(1) $$P_{z}=\frac{1}{S}\int z\left(\rho_{\mathrm{ions}}+\rho_{\mathrm{valence}}\right)d^{3}r$$

where *S* denotes the area of the unit cell, ρ_ions_ and ρ_valence_ represent the charge densities of the ions and valence electrons, respectively. Based on this definition, the out‐of‐plane ferroelectric polarization *P_z_* is estimated to be 2.46 pC m^−1^. Notably, this polarization magnitude exceeds those reported for several previously studied 2D FE systems, such as 1T'‐ReS_2_ (0.07 pC m^−1^) [32] and Hf_2_VC_2_F_2_ (0.29 pC m^−1^) [33]. Considering that electric polarization has been experimentally detected in 2D 1T'‐ReS_2_, it should therefore be feasible to experimentally measure the FE polarization in Cu‐BiCl_3_.

#### 2.2.2 Topological Metallic States in Monolayer Cu‐BiCl_3_

**FIGURE 3:**
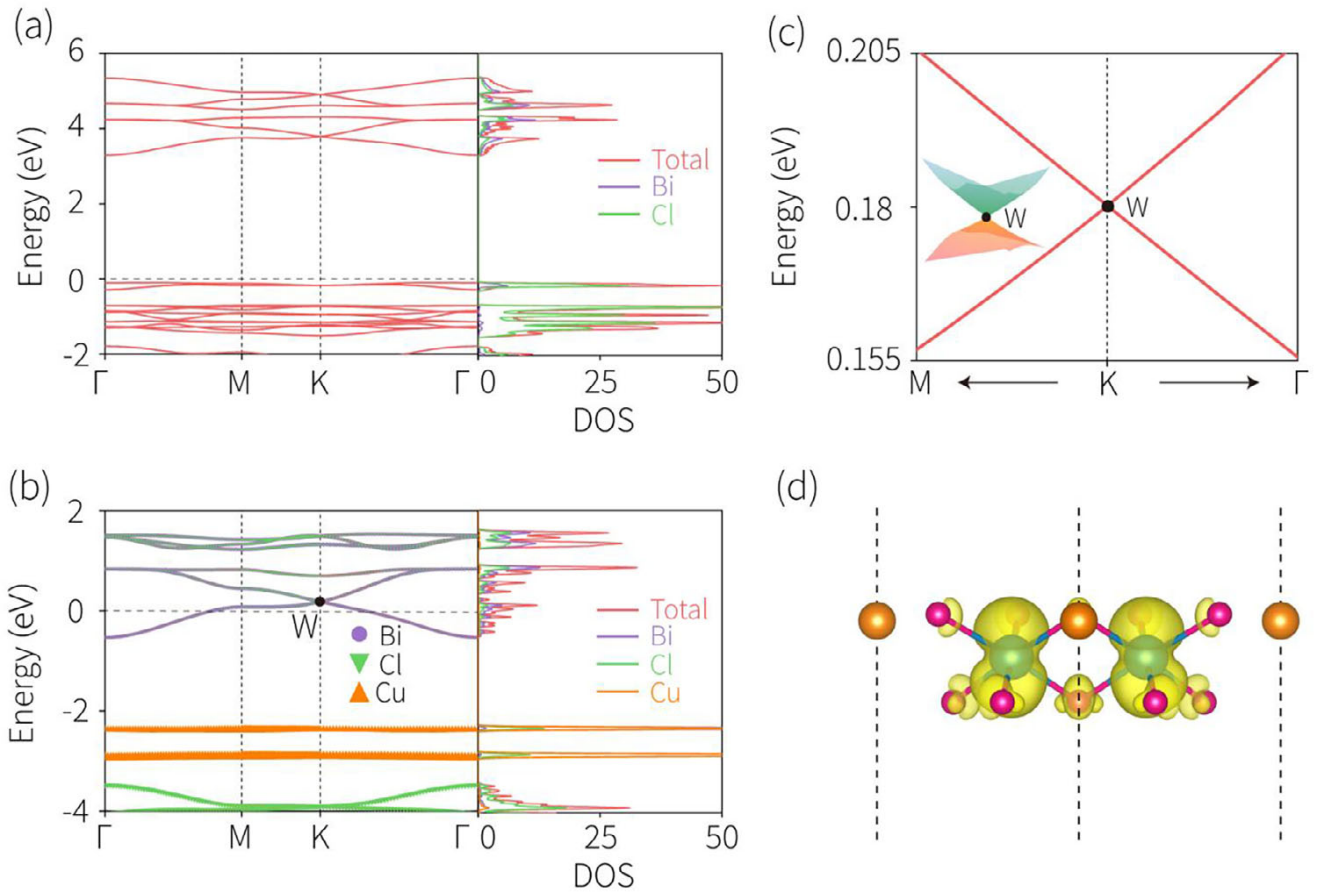
(a,b) Electronic band structures of BiCl_3_ and Cu‐BiCl_3_, respectively. (c) Zoomed‐in band structure around the Weyl point (W). The inset depicts the 3D band dispersion of the Weyl point (W). (d) Partial charge density near the Weyl point in the vicinity of the Fermi level.

As shown in Figure 3a, pristine monolayer BiCl_3_ exhibits characteristic semiconducting behavior. Upon Cu intercalation, the electronic structure is substantially reconstructed, as illustrated in Figure 3b. The intercalated Cu strongly hybridizes with the surrounding Cl atoms, leading to a pronounced enhancement of the density of states near the Fermi level and ultimately driving a semiconductor‐to‐metal transition. This metallization provides an electronic basis for efficient interfacial charge transfer and lays the groundwork for activating the otherwise inert basal‐plane environment.

Notably, within the reconstructed metallic band structure, band‐crossing points emerge in the vicinity of the Fermi level, corresponding to the appearance of Weyl points at the K and K′ high‐symmetry points. We used IRVSP [34, 35] to analyze the band representations (BRs) at the band crossing at the high‐symmetry K point in the Cu‐AB_3_ monolayers, both without and with spin‐orbit coupling. The corresponding irreducible‐representation labels have been added to Figures S6 and S7. The results show that the crossing remains twofold degenerate in both cases. The Weyl points at the K and K′ points exhibit opposite quantized Berry phases and associated edge states, supporting their nontrivial topological character (see Figures S8 and S9).

The 3D linear dispersion of the Weyl point (at the K point) is shown in Figure 3c, where a clear twofold degeneracy is observed. From the two linear bands near the Fermi level E_f_, the Fermi velocity 𝑣_𝐹_ of the charge carriers can be evaluated using linear fitting according to

(2) $$\nu_{F}=\frac{1}{\hbar }\;\frac{\mathrm{dE}}{\mathrm{dk}}\;$$

Using this relation, we obtain 𝑣_𝐹_ ≈ 0.368 × 10^6^ m s^−1^, which is on the same order of magnitude as that of graphene (≈ 1.0 × 10^6^ m s^−1^) [36].

#### 2.2.3 Catalytically Active Sites and Controllable HER Activity in Monolayer Cu‐BiCl_3_

To quantify the catalytic performance, a 2 × 2 supercell was constructed and four possible H* adsorption sites were evaluated (Figure S10). The calculated Gibbs free energies of H* adsorption (Δ*G*_H*_) for Cu–BiCl_3_ in the two FE states are summarized in Table S3. Evidently, Cu intercalation leads to a substantial improvement in Δ*G*_H*_. Among them, the D2 site, located at the top of Bi atom, exhibits the highest catalysis activity (Δ*G*_H*_ ≈ ‐0.02 eV). This preference originates from its distinct electronic structure. The emergence of Weyl points with high charge carriers around the Fermi level significantly enhances the electronic mobility, which is expected to facilitate charge transport during HER [37, 38, 39, 40]. Moreover, the partial charge density associated with the Weyl points is predominantly localized on the Bi atoms after Cu intercalation (see Figure 3d), rendering the D2 site (at the top of Bi atom) the most favorable adsorption site for H*. Consequently, the D2 site offers the most suitable electronic environment for stabilizing the H*, leading to superior HER performance.

**FIGURE 4:**
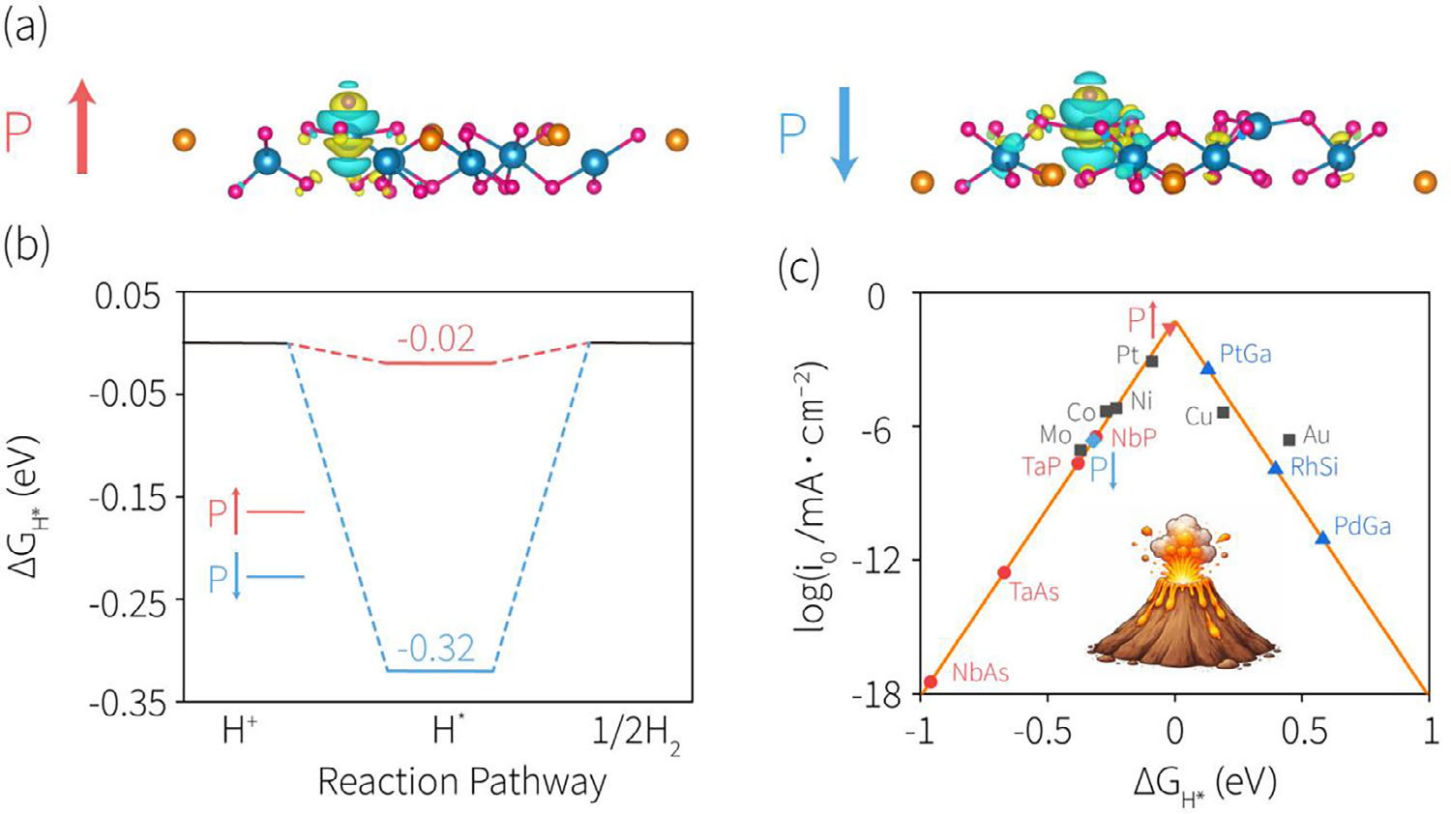
(a) Charge‐density difference after H adsorption on Cu–BiCl_3_ in the P↑ and P↓ FE states. (b) ΔG_H*_ for Cu–BiCl_3_ in the P↑ and P↓ FE states. (c) HER volcano plot of Cu–BiCl_3_ in the P↑ and P↓ FE states, compared with representative HER catalysts. Data for the reference catalysts are taken from refs. [38, 49, 50].

Beyond the role of electronic topology, ferroelectricity offers an additional and powerful degree of freedom for regulating surface charge distribution and catalytic activity. FE catalysts with switchable polarization have been widely demonstrated to modulate charge distribution and local electronic states, thereby altering adsorbate binding and catalytic activity [41, 42, 43, 44, 45, 46, 47]. Here, we explicitly demonstrate such a polarization‐dependent catalytic behavior in Cu‐intercalated BiCl_3_. As shown in Figure 4b, the two FE polarization states exhibit distinctly different HER activities. In the P↑ state, H* adsorption is thermodynamically favorable, with Δ*G*_H*_ ≈ −0.02 eV, which is close to the optimal value for HER (Δ*G*_H*_ = 0 eV). In contrast, the P↓ state binds H* much more strongly, yielding a significantly more negative adsorption free energy (Δ*G*_H*_ ≈ −0.32 eV). The P↑ state is positioned near the apex of the HER volcano plot (Figure 4c), signifying an optimal balance between adsorption strength and reaction kinetics, and yielding a catalytic performance comparable to that of typical high‐performance HER catalysts.

The Bader‐charge analysis (see Table S4) shows that the amount of charge gained by the adsorbed H atom remains very similar in the two FE states, indicating that the distinct hydrogen adsorption behavior cannot be simply attributed to the final net charge accumulated on H. Meanwhile, the charge‐density‐difference plots reveal a consistent charge‐transfer pattern upon H adsorption in both polarization states: electron density is mainly depleted from the neighboring Bi atoms and accumulates around the adsorbed H atom. This indicates that the Bi‐top site serves as the primary electron source for stabilizing H atom on the basal plane (see Figure 4a). Therefore, the key difference between the two FE states is more reasonably understood not in terms of the final amount of charge gained by H, but in terms of the polarization‐induced modulation of the local surface electronic environment.

The work function, defined as the minimum energy required to remove an electron from the Fermi level of a solid to the vacuum level, is a widely used descriptor of surface electronic properties and electron‐transfer capability. Because catalytic reactions are closely associated with interfacial charge redistribution and electron exchange between the surface and adsorbates, changes in the work function can offer valuable insight into variations in catalytic behavior [48]. Previous study on FE material BaTiO_3_ has shown that polarization switching can significantly modify the surface electronic states and work function, thereby changing the HER activity [46]. Similarly, for the Cu‐AB_3_ monolayers, polarization reversal changes the surface work function, and the lower‐work‐function FE state shows more favorable H adsorption energetics and a smaller HER reaction barrier. Therefore, the polarization‐induced change in the work function provides a reasonable explanation for the distinct hydrogen adsorption behavior and HER activity observed in the two FE states.

As shown in Table S5, the work function of Cu‐BiCl_3_ changes from 4.1552 eV in the P↑ state to 4.431 eV in the P↓ state, indicating that polarization reversal significantly modifies the surface electronic environment. Since FE polarization affects the surface dipole and electrostatic potential, such a change in work function is physically expected. This variation is consistent with the distinct hydrogen adsorption thermodynamics in the two FE states, thereby establishing a direct link between polarization reversal, work‐function modulation, and HER activity.

### 2.3 Linear Correlation between FE Polarization and HER Activity of Cu‐AB_3_

**FIGURE 5:**
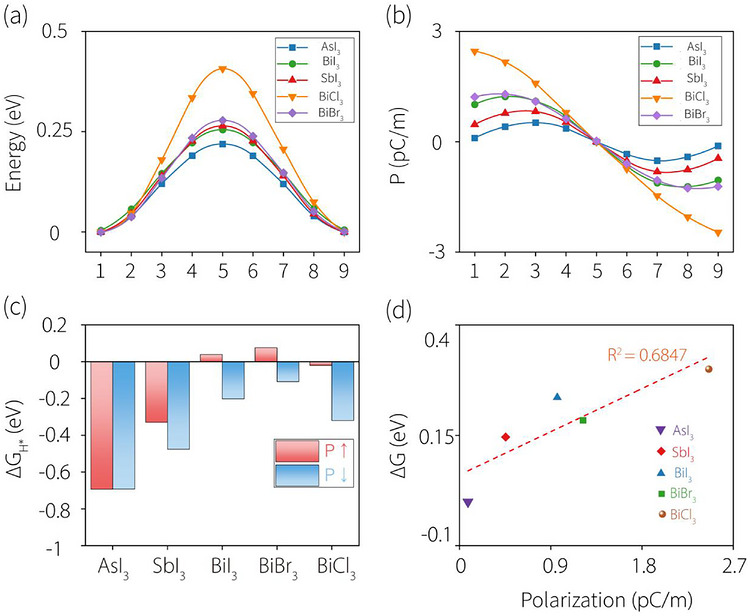
(a) FE switching energy barrier of Cu‐AB_3_. (b) Evolution of FE polarization along the FE switching pathway in Cu‐AB_3_ monolayers. (c) Calculated Δ*G*_H*_ of Cu‐AB_3_ monolayers in the two FE states. (d) Correlation between the calculated ΔG and FE polarization of Cu–AB_3_ monolayers.

To establish a general and quantitative understanding of polarization‐controlled catalysis beyond the representative Cu–BiCl_3_, we first analyze the FE switchability across the Cu‐AB_3_ family. As shown in Figure 5a, all considered Cu‐AB_3_ monolayers exhibit finite yet moderate FE switching barriers, ranging from 0.22 to 0.41 eV, indicating that polarization reversal is energetically accessible. Consistently, the polarization evolves continuously along the switching pathway (Figure 5b), confirming a robust and reversible FE order in these systems. Taken together, the switching barriers and polarization evolution demonstrate that Cu‐AB_3_ monolayers provide a FE platform in which surface charge distribution can be deterministically reconfigured via FE polarization switching.

Having established the switchability of ferroelectricity in Cu‐AB_3_ monolayers, we next examine how polarization reversal translates into changes in the hydrogen adsorption process. Figure 5c summarizes the calculated Δ*G*_H*_ for Cu‐AB_3_ monolayers in the two FE polarization states, clearly demonstrating that polarization reversal systematically modulates hydrogen adsorption activity. To quantitatively assess the difference in catalytic activity between the two FE states, we define ΔG as the difference in ΔG_H*_ between the P↑ and P↓ states (Δ*G* = Δ*G*_H*(P↑)—_ΔG_H*(P↓)_). In Figure 5d, we establish a linear dependence between FE polarization and ΔG: a larger polarization for the P↑ and P↓ states leads to a larger Δ*G*. For example, in Cu‐AsI_3_, the FE polarization is relatively small (0.08 pC m^−1^), resulting in a Δ*G* value that is also close to zero (0.0007 eV), indicating that polarization reversal has little effect on tuning its HER activity. In contrast, Cu‐BiCl_3_ exhibits a much larger FE polarization (2.46 pC m^−1^), accompanied by a correspondingly large Δ*G* (0.30 eV). In this case, polarization reversal significantly amplifies interfacial charge redistribution, leading to a pronounced modulation of hydrogen adsorption thermodynamics. Consequently, FE switching provides a deterministic and reversible handle to navigate H* adsorption toward or away from the optimal condition, offering a practical route for electrically controllable HER activity in Cu‐AB_3_ monolayers.

The difference in hydrogen adsorption behavior between the two FE states can also be rationalized in terms of the work function. In general, for two FE states, a larger work function implies less favorable electron transfer from the surface to the adsorbate, which can weaken the H–surface interaction and thus contribute to a larger Δ*G*_H*_. Therefore, the polarization‐induced variation in the work function provides a reasonable explanation for the distinct hydrogen adsorption behavior and HER activity observed in the two FE states. Consistently, Figure S11 shows that ΔG is positively correlated with Δ*ϕ*, and this trend is in line with the dependence of Δ*G* on polarization in Figure 5d. This consistency supports a close correlation between polarization, work‐function variation, and hydrogen adsorption behavior.

Before closing this work, we note the following points: (i) Considerable efforts have long been devoted to the pursuit of FE metals—a class of materials that simultaneously host spontaneous electric polarization and metallic conductivity, two properties that are seemingly incompatible. In 2D materials, conduction electrons are confined within the plane, which allows external electric fields to penetrate the material and reverse the out‐of‐plane polarization [51, 52]. From this perspective, single‐atom intercalation in 2D systems provides a physically sound and effective route to concurrently drive semiconductor‐to‐metal transitions and nonpolar‐to‐polar layer‐group transformations, ultimately enabling the realization of FE metals. (ii) Unlike conventional wide‐bandgap ferroelectrics, FE metals naturally host electronic states at the Fermi level, which enables the emergence of quantum states and endows them with additional topological properties. Although FE materials have been demonstrated to be beneficial for catalysis, most current studies of FE materials are concentrated in semiconducting or insulating systems [53, 54, 55], whereas catalytic functionalities in metallic ferroelectrics remain largely unexplored. (iii) Metallic Weyl‐point states can intrinsically enhance catalytic activity by providing a high density of itinerant carriers. Moreover, switching the FE polarization offers an additional knob to reversibly reshape the surface charge distribution and the local electronic environment at the active sites. Together, the Weyl‐like band crossing around the Fermi level and FE polarization switching constitute a dual‐optimization strategy that simultaneously boosts basal‐plane catalysis and enables deterministic, electrically controllable tuning of the reaction thermodynamics. Consequently, the integration of topology, metallicity, ferroelectricity, and catalysis establishes a highly interdisciplinary platform, opening new avenues for cross‐fertilization between condensed‐matter physics, materials science, and electrocatalysis.

## 3 Conclusion

In conclusion, we demonstrate that single‐atom intercalation provides a unified strategy to activate the basal‐plane HER activity in porous AB_3_ monolayers by simultaneously reconstructing lattice symmetry and electronic states. Taking Cu–BiCl_3_ as a representative example, Cu intercalation drives a semiconductor–metal transition and introduces Weyl band crossings near the Fermi level, while the associated electronic states are mainly localized around Bi atoms. Importantly, the intercalation‐induced FE polarization enables deterministic and reversible regulation of adsorption thermodynamics through modulation of the local surface electronic environment, as also reflected by the work‐function variation. We further find a linear correlation between FE polarization and ΔG for Cu–AB_3_, with larger polarization yielding a larger ΔG. To our knowledge, this work represents the first attempt to integrate topology, metallicity, ferroelectricity, and catalysis within a single materials platform, opening a new route toward topological FE metal catalysts.

## Conflicts of Interest

The authors declare no conflicts of interest.

## Supporting information




**Supporting File**: advs75741‐sup‐0001‐SuppMat.pdf.

## Data Availability

The data that support the findings of this study are available from the corresponding author upon reasonable request.
