# Supplementary material for: Single‐Atom Intercalation‐Driven Topological Ferroelectric Metal for High‐Performance Hydrogen Evolution Reaction
Source: Adv Sci (Weinh). 2026 May 19:e75741. Online ahead of print. doi: 10.1002/advs.75741 (PMC13336059; doi:10.1002/advs.75741)
Supplement: Supplementary file 1 — Supporting File: advs75741‐sup‐0001‐SuppMat.pdf. [file ADVS-9999-e75741-s001.pdf]

Supporting Information for

Single-Atom Intercalation–Driven Topological Ferroelectric Metal for High-Performance Hydrogen Evolution Reaction

Rongxuan Lu, Jian Zhang, Jialin Gong, Wenxian Li*, Gang Zhang**

R. Lu, W. Li
School of Chemical Engineering,
University of New South Wales,
Sydney, New South Wales, 2052 Australia
*E-mail: wenxian.li1@unsw.edu.au

J. Gong
Institute for Superconducting and Electronic Materials,
Faculty of Engineering and Information Sciences,
University of Wollongong,
Wollongong, New South Wales 2500, Australia
*E-mail: jg426@uowmail.edu.au

J. Zhang, G. Zhang
Yangtze Delta Region Academy in Jiaxing, Beijing Institute of Technology, Jiaxing 314019, China
School of Materials Science and Engineering, Beijing Institute of Technology, Beijing 100081, China
*E-mail: gangzhang@bit.edu.cn

Keywords: Single-atom intercalation, ferroelectric Weyl metal, HER

S1. Computational Methods

The first-principles calculations were performed in the framework of DFT within the Vienna Ab initio simulation package (VASP)[1,2]. The exchange-correlation effect was treated in the GGA of the Perdew-Burke-Ernzerhof (PBE) function[3]. To take into account the weak van der Waals interactions, the DFT-D3 method with the Becke–Johnson damping scheme[4] was adopted. To account for strong correlation effects relevant to the Cu-3*d* orbitals, the Hubbard *U* correction via the *GGA+U* approach was used. The value of the *U* is set as 4.0 eV, as reported in Ref. [5]. An over 15-Å thick vacuum layer was used to ensure decoupling between neighboring slabs. All calculations were performed with a plane-wave cutoff of 500 eV, and the convergence criterion for the electronic self-consistence loop was set to be 10^{-6} eV on the 9 ×9×1 *k*-point mesh, and for the structural relaxation, the Hellmann-Feynman force on each atom was taken to be -0.01 eV $Å^{-1}$. The ab initio molecular dynamics (AIMD) simulations were carried out by using 2 × 2 supercells at 300 K within each time step of 9 ps. The phonon spectra were calculated using the PhononBench[6-9] platform which is a web-based service for efficient phonon-spectrum calculations and dynamical-stability assessment of crystalline materials. We note that, among the investigated systems, only Cu-SbI_3 exhibits tiny imaginary frequencies near the Γ point in the low-frequency acoustic branches; these are likely numerical artifacts rather than evidence of substantive dynamical instability, and similar features are also commonly seen in other reported two-dimensional materials.[10,11] The edge states and Berry curvature are calculated using the WannierTools package.[12]

The catalytic performance is commonly quantified using the Gibbs free energy of hydrogen adsorption (ΔG_{H^*}), defined as:

$$\triangle G_{H^*} = \triangle E_H + \triangle E_{ZPE} - T \triangle S_H, \quad (1)$$

where ΔE_H represents the hydrogen adsorption energy on the substrate and is defined as:

$$\triangle E_H = (E_{Cu-AB_3+H} - E_{Cu-AB_3} - \frac{1}{2} E_{H_2}), \quad (2)$$

where E_{Cu-AB_3+H} and E_{Cu-AB_3} denote the total energies of hydrogen adsorbed system and the pristine substrate, respectively. E_{H_2} is the total energy of the hydrogen molecule. Besides, $\triangle E_{ZPE}$[13] is the difference of the zero-point energy between adsorbed and gaseous hydrogen. T is the surface temperature, which is taken to be room temperature (T = 300 K). $\triangle S_H$[14,15] is the corresponding entropy change, as given by:

$$\triangle S_H = -\frac{1}{2} S^0_{H_2}. \quad (3)$$

Finally, Eq. (1) can be simplified as:

$$\triangle G_{H^*} = \triangle E_H + 0.22 \text{ eV}. \quad (4)$$

References

[1] G. Kresse and J. Hafner, "Ab initio molecular-dynamics simulation of the liquid-metal-amorphous-semiconductor transition in germanium," *Physical Review B* **49** (1994): 14251.

[2] G. Kresse and J. Furthmüller, "Efficient iterative schemes for ab initio total-energy calculations using a plane-wave basis set," *Physical Review B* **54** (1996): 11169.

[3] J. P. Perdew, K. Burke, and M. Ernzerhof, "Generalized gradient approximation made simple," *Physical Review Letters* **77** (1996): 3865.

[4] S. Grimme, "A general quantum mechanically derived force field (QMDFF) for molecules and condensed phase simulations," *Journal of Chemical Theory and Computation* **10** (2014): 4497–4514.

[5] L. Wang, T. Maxisch, and G. Ceder, "Oxidation energies of transition metal oxides within the GGA + U framework," *Physical Review B* **73** (2006): 195107.

[6] X.-Q. Han, P.-J. Guo, Z.-F. Gao, and Z.-Y. Lu, "PhononBench: A large-scale phonon-based benchmark for dynamical stability in crystal generation," *arXiv* (2025): arXiv:2512.21227.

[7] X.-Q. Han, X.-D. Wang, M.-Y. Xu, Z. Feng, B.-W. Yao, P.-J. Guo, Z.-F. Gao, and Z.-Y. Lu, "AI-Driven inverse design of materials: past, present, and future," *Chinese Physics Letters* **42** (2025): 027403.

[8] X.-Q. Han, P.-J. Guo, Z.-F. Gao, H. Sun, and Z.-Y. Lu, "InvDesFlow-AL: active learning-based workflow for inverse design of functional materials," *npj Computational Materials* **11** (2025): 364.

[9] Z. Ouyang, B. W. Yao, X. Q. Han, W. Chen, X. Huang, D. Semenok, A. G. Kvashnin, and T. Cui, "High-temperature superconductivity in Li_2AuH_6 mediated by strong electron-phonon coupling under ambient pressure," *Physical Review B* **111** (2025): L140501.

[10] R.-W. Zhang, C. Cui, R. Li, J. Duan, L. Li, Z.-M. Yu, and Y. Yao, "Predictable gate-field control of spin in altermagnets with spin-layer coupling," *Physical Review Letters* **133** (2024): 056401.

[11] F. Iyiakanat, E. Sari, and H. Sahin, "Thinning $CsPb_2Br_5$ perovskite down to monolayers: Cs-dependent stability," *Physical Review B* **96** (2017): 155442.

[12] Q. Wu, S. Zhang, H.-F. Song, M. Troyer, and A. A. Soluyanov, "WannierTools: an open-source software package for novel topological materials," *Computer Physics Communications* **224** (2018): 405–416.

[13] J. K. Nørskov, T. Bligaard, A. Logadottir, J. R. Kitchin, J. G. Chen, S. Pandelov, and U. Stimming, "Trends in The exchange current for hydrogen evolution," *Journal of The Electrochemical Society* **152** (2005): J23-J26.

[14] L. Li, J. Zeng, W. Qin, P. Cui, and Z. Zhang, "Tuning the hydrogen activation reactivity on topological insulator heterostructures," *Nano Energy* **58** (2019): 40–46.

[15] Y. An, X. Fan, H. Liu, and Z. Luo, "Improved catalytic performance of monolayer nano-triangles WS_2 and MoS_2 on HER by 3d metals doping," *Computational Materials Science* **159** (2019): 333–340.

[16] J. Li, Y. Liu, J. Bai, C. Xie, H. Yuan, Z. Cheng, W. Wang, X. Wang, and G. Zhang, "Phononic weyl pair, phononic weyl complex, phononic real chern insulator state, and phononic corner modes in 2D Kekulé-order graphene," *Applied Physics Reviews* **10** (2023): 031416.

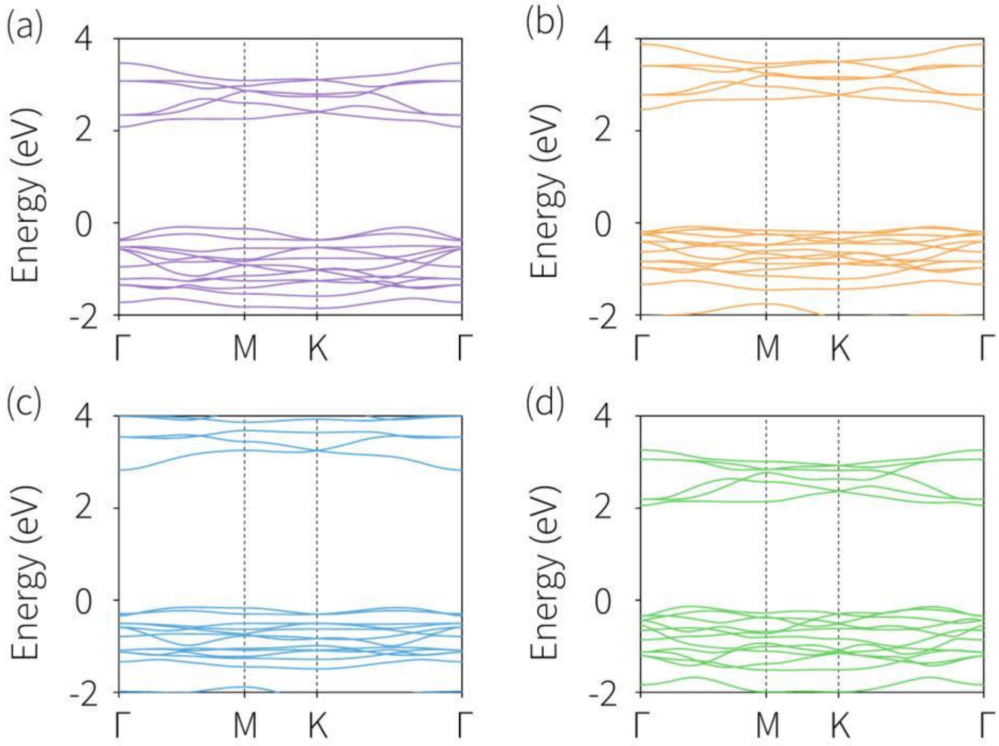

Figure S1. (a-d) Band structures of SbI_3, BiI_3, $BiBr_3$, AsI_3 monolayers, respectively.

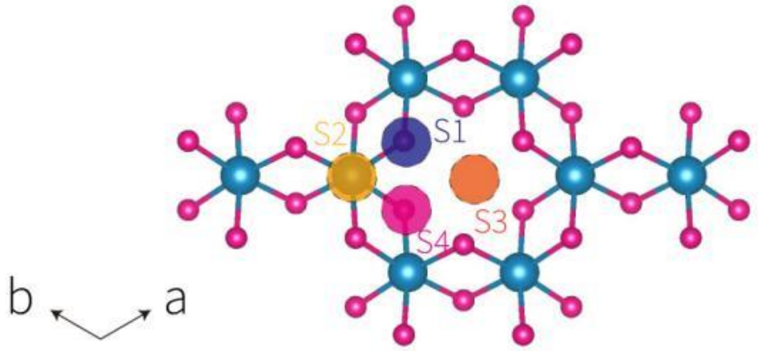

Figure S2. Adsorption sites of H* at AB_3 monolayers.

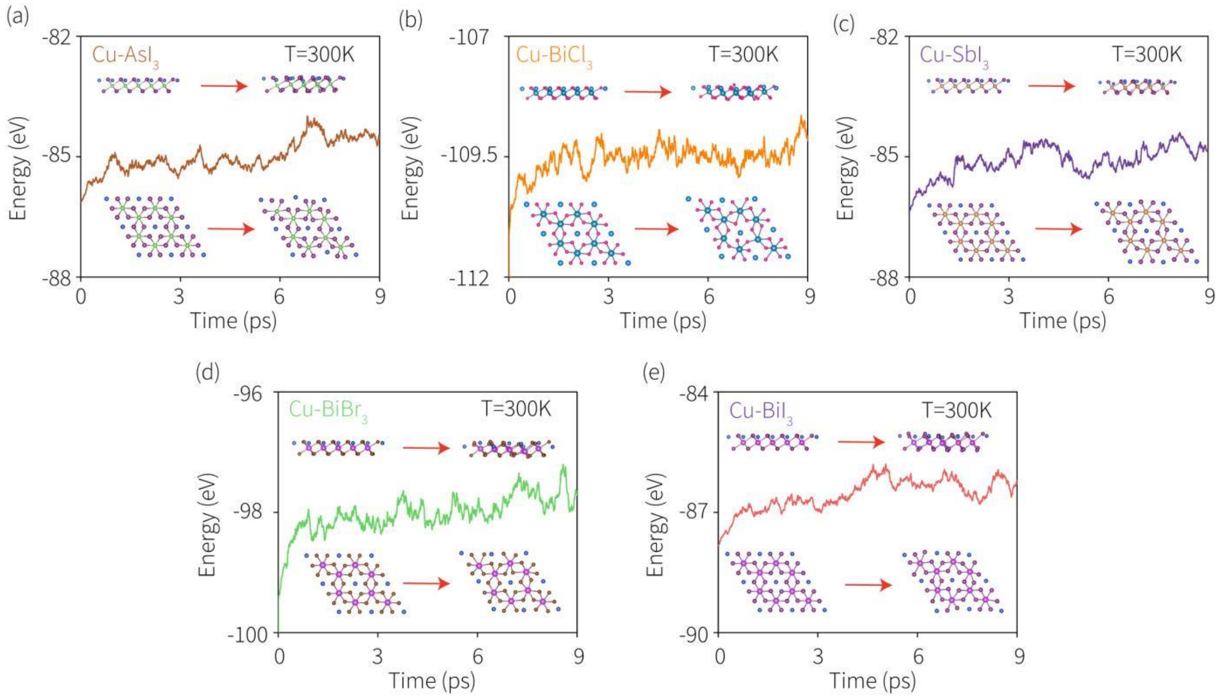

Figure S3. (a)-(e) Ab initio molecular dynamics (AIMD) simulations of Cu-AB_3 monolayers.

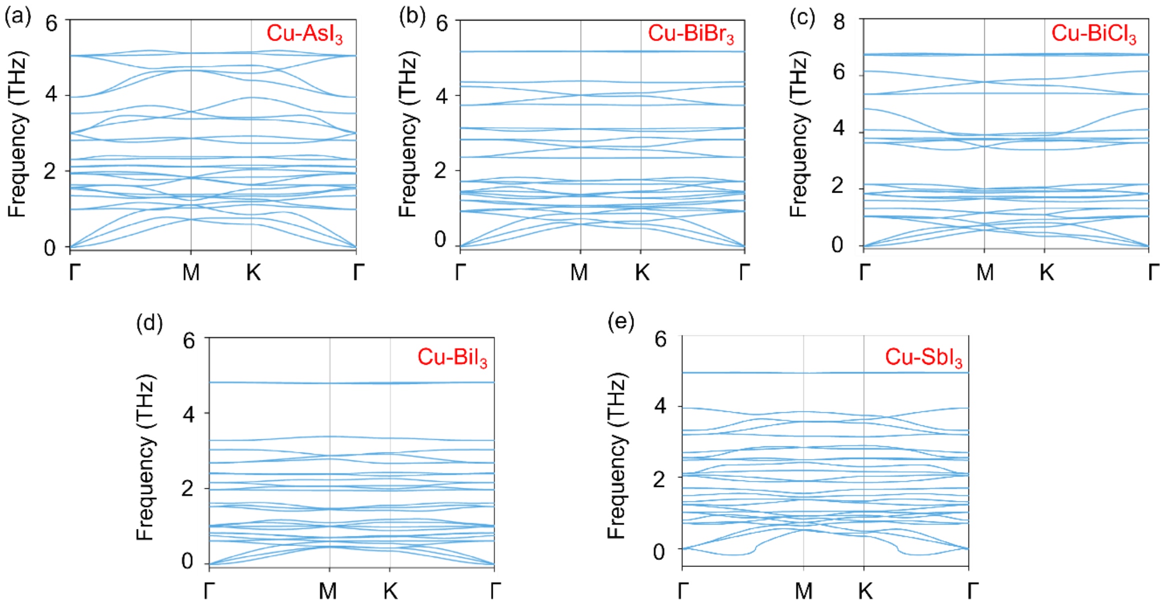

Figure S4. Phonon spectra of the Cu-AB_3 monolayers obtained using the PhononBench online platform.

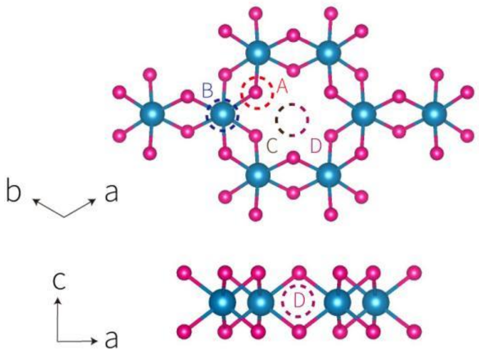

Figure S5. The possible adsorption sites for a Cu atom on the $BiCl_3$ monolayer.

Table S1. Calculated ΔG_{H^*} (eV) of AB_3 monolayers at different adsorption sites.

	S1	S2	S3	S4
AsI_3	1.817	1.673	2.455	2.552
SbI_3	1.900	1.958	2.564	2.343
BiI_3	2.046	2.065	2.624	2.500
$BiBr_3$	1.900	2.591	2.741	2.691
$BiCl_3$	1.740	2.829	2.861	2.848

Table S2. Criteria to identify the most suitable intercalation sites in AB_3 monolayer. **Criteria 1**: Cu atom should steadily adsorbed on the AB_3 monolayers surfaces. **Criteria 2:** the favorable adsorption site should be able to induce polarization switching. **Criteria 3:** after the intercalation, the Cu-AB_3 monolayers should be able to activate H* and maintain the ferroelectricity. The adsorption sites that satisfy the criteria are highlighted in red. The adsorption energy (E_{ads}) of Cu-Intercalation is defined as $E_{ads} = E_{Cu-AB_3} - (E_{Cu} + E_{AB_3})$. where E_{Cu-AB_3}, E_{Cu} , and E_{AB_3} represent the total energy of Cu-AB_3, the Cu single atom and AB_3 monolayers, respectively.

	**Criteria 1**	**Criteria 2**	**Criteria 3**
A	Yes	Yes	No
B	Yes	No	--
C	Yes	Yes	Yes
D	Yes	No	--

"Yes" indicates that the Cu atom adsorption satisfies the screening criteria.

"No" indicates that the Cu atom adsorption fails to satisfy the screening criteria.

"--" indicates that these Cu adsorption configurations were not considered in the third screening procedure, because they did not satisfy Criterion 2.

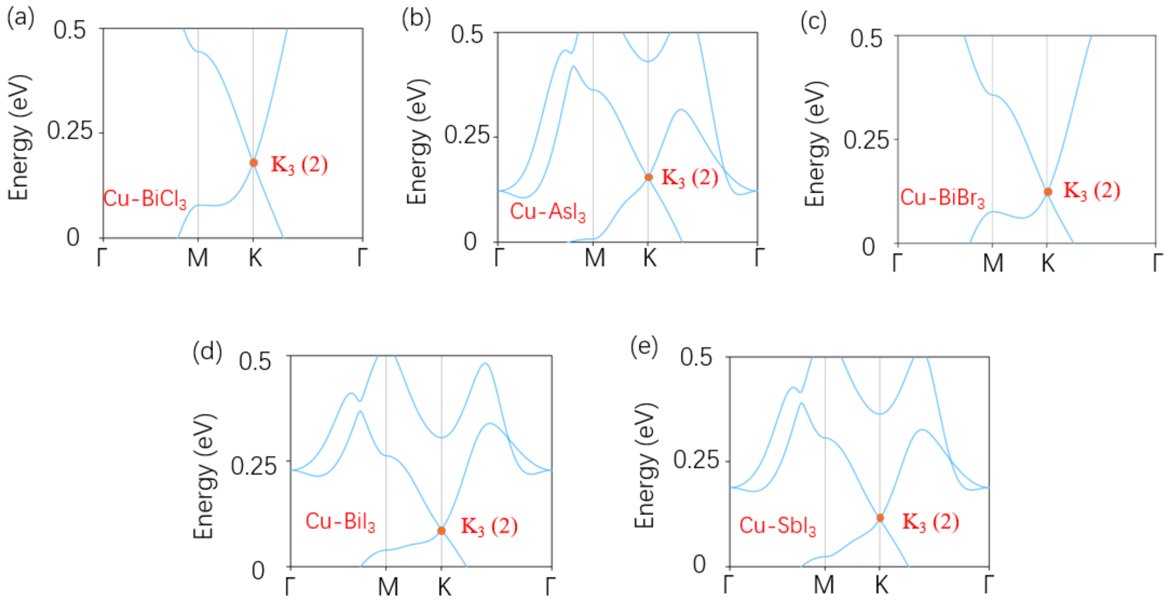

Figure S6. Calculated band structures of the Cu-AB3 monolayers without SOC. The band representations (BRs) of the Weyl point at the high-symmetry K point are labeled.

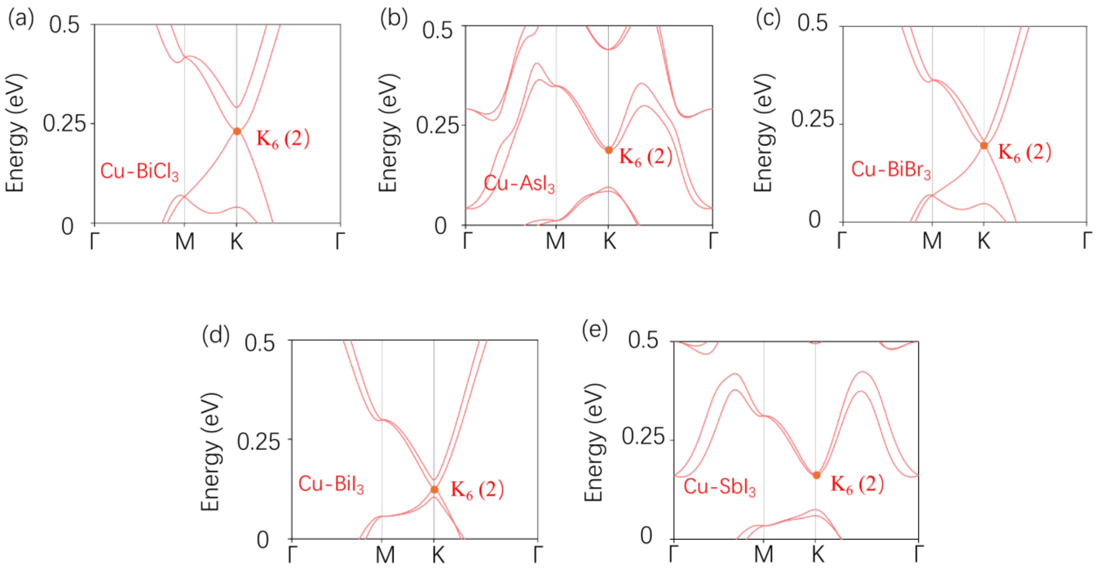

Figure S7. Calculated band structures of the Cu-AB3 monolayers with SOC. The band representations (BRs) of the Weyl point at the high-symmetry K point are labeled.

Note that in Cu-AB_3 monolayers, the crossing points at the K and K′ high symmetry points (HSPs) appear in pairs, and the crossing points pair can be characterized by the Berry phase, which is defined based on a small circle enclosing the crossing points at the K and K′ HSPs:

$$\gamma_B = \oint_{\ell} A(k)dk, mod 2\pi, \quad (5)$$

where $A(k)$ is the Berry connection for the Bloch-like wave function.[16]

As shown in Figures S8(a)–(e), the Weyl points at the K and K′ high-symmetry points are characterized by quantized Berry phases of opposite sign, with one being $+\pi$ and the other $-\pi$. Furthermore, we performed edge-state calculations and identified edge states arising from the projections of the Weyl points along the (100) direction, as shown in Figure S9. These results further support the nontrivial topological nature of the Cu-AB_3 monolayers.

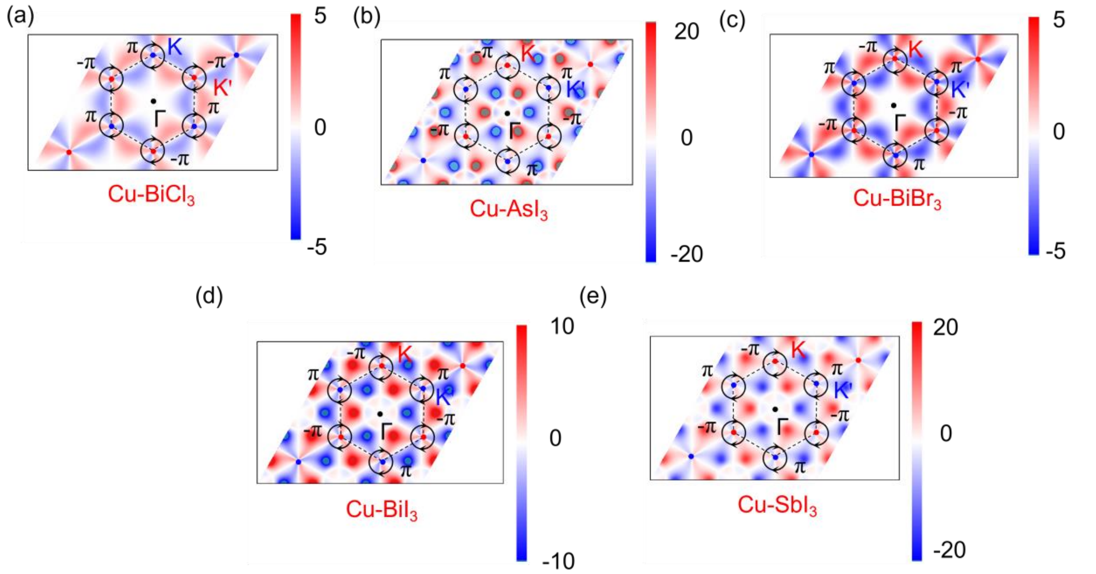

Figure S8. (a-e) Berry curvature distributions for Weyl points at K and K' HSPs. We used dotted lines to show the first BZ. The Berry phases around the K and K′ valleys are quantized with opposite signs, i.e., one is $+\pi$ and the other is $-\pi$.

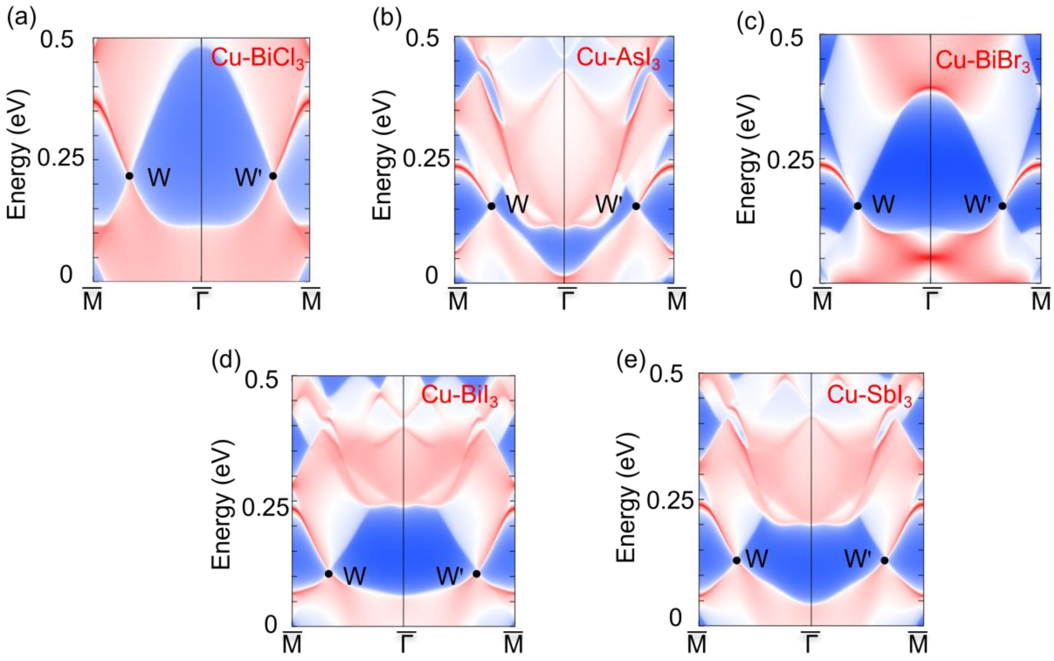

Figure S9. (a-e) Calculated edge states of Cu-AB_3 along (100) direction.

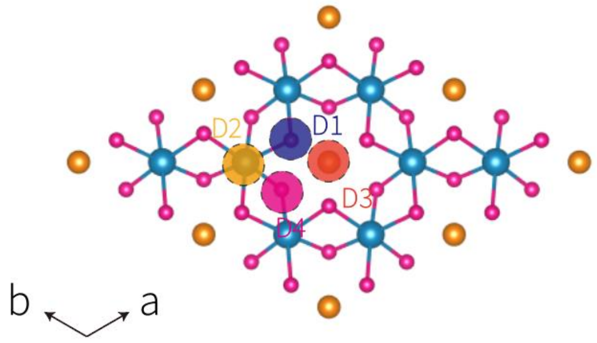

Figure S10. Adsorption sites of H* at Cu-$BiCl_3$ monolayer.

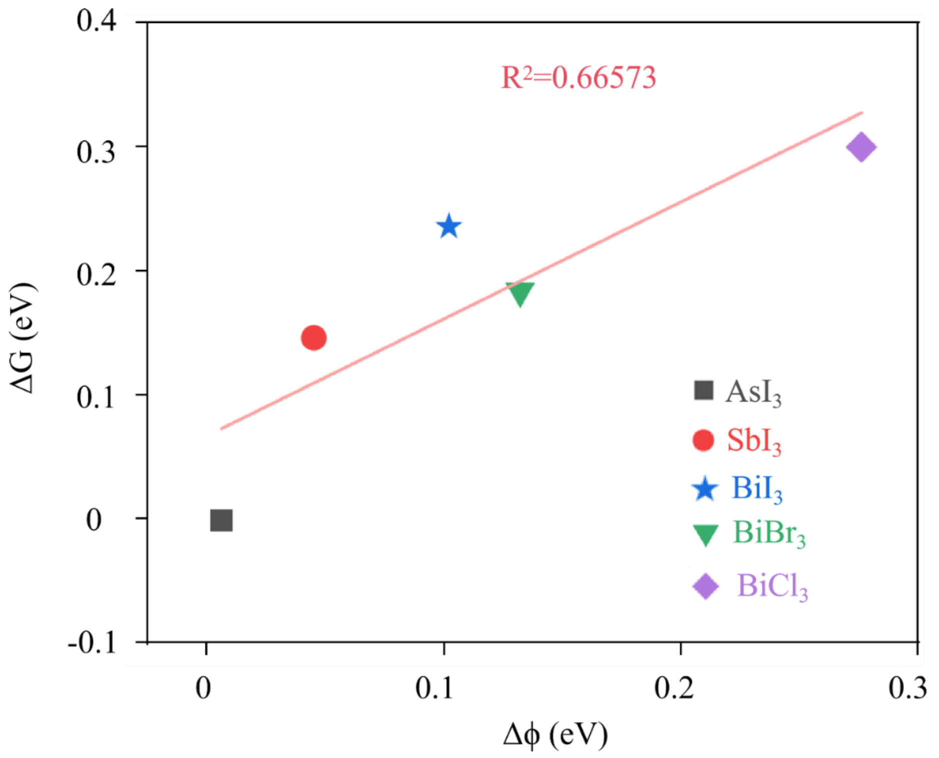

Figure S11. Linear fitting of ΔG as a function of $\Delta\phi$.

Table S3. Calculated ΔG_{H^*} (eV) for the H* adsorption sites in Cu-$BiCl_3$ monolayer under different FE states.

D1		D2		D3		D4	
P↑	P↓	P↑	P↓	P↑	P↓	P↑	P↓
-0.64	-0.43	-0.02	-0.32	1.10	0.93	-1.22	-0.58

Table S4. Charge gained by the adsorbed H atom on Cu-AB_3 monolayers.

Materials	Charge (e)	Materials	Charge (e)
Cu-$BiCl_3$ (P ↑)	0.2546	Cu-$BiCl_3$ (P ↓)	0.2648
Cu-BiI_3 (P ↑)	0.2451	Cu-BiI_3 (P ↓)	0.2511
Cu-$BiBr_3$ (P ↑)	0.2648	Cu-$BiBr_3$ (P ↓)	0.2485
Cu-AsI_3 (P ↑)	0.2651	Cu-AsI_3 (P ↓)	0.2488
Cu-SbI_3 (P ↑)	0.2615	Cu-SbI_3 (P ↓)	0.2517

Table S5. Work functions (ϕ) of Cu-AB_3 monolayers in two ferroelectric states.

Materials	Work function (eV)	Materials	Work function (eV)	$\Delta\phi$
Cu-$BiCl_3$ (P ↑)	4.1552	Cu-$BiCl_3$ (P ↓)	4.431	0.27
Cu-BiI_3 (P ↑)	3.888	Cu-BiI_3 (P ↓)	3.990	0.10
Cu-$BiBr_3$ (P ↑)	4.136	Cu-$BiBr_3$ (P ↓)	4.268	0.13
Cu-AsI_3 (P ↑)	4.071	Cu-AsI_3 (P ↓)	4.077	0.006
Cu-SbI_3 (P ↑)	3.980	Cu-SbI_3 (P ↓)	4.025	0.045
